# Demographic and Injury Characteristics as Potential Risk Factors for Anterior Cruciate Ligament Injuries: A Multicentric Cross-Sectional Study

**DOI:** 10.3390/jcm13175063

**Published:** 2024-08-27

**Authors:** Mehdi Motififard, Hossein Akbari Aghdam, Hadi Ravanbod, Mohammad Saleh Jafarpishe, Mahdi Shahsavan, Amin Daemi, Amir Mehrvar, Arghavan Rezvani, Hossein Jamalirad, Mahdie Jajroudi, Mohammad Shahsavan

**Affiliations:** 1 Department of Orthopedic Surgery, Isfahan University of Medical Sciences, Isfahan 81746-73461, Iran; 2 Department of Radiology, Isfahan University of Medical Sciences, Isfahan 81746-73461, Iran; 3 Department of Medical Biochemistry, Faculty of Medicine, Cukurova University, Adana 01330, Turkey; phd_bio@yahoo.com; 4 Department of Orthopedic Surgery, Shahid Beheshti University of Medical Sciences, Tehran 19839-69411, Iran; 5 Department of Medical Informatics, Mashhad University of Medical Sciences, Mashhad 91779-48564, Iran; 6 Pharmaceutical Research Center, Mashhad University of Medical Sciences, Mashhad 91779-48564, Iran

**Keywords:** anterior cruciate ligament, ACL injury, sports injury, risk factors, injury mechanism, injury prevention

## Abstract

**Background:** Anterior cruciate ligament (ACL) injuries are prevalent and can have debilitating consequences, with various factors potentially influencing their occurrence. This multicentric study aimed to comprehensively analyze the epidemiological characteristics of ACL injuries. We hypothesized that specific patient characteristics, such as age, sex, body mass index (BMI), and sports involvement, would be associated with distinct injury patterns and risk profiles. **Methods:** This cross-sectional study analyzed the medical records of 712 patients aged 15–60 diagnosed with ACL rupture. Data on demographics, injury mechanisms, associated injuries, graft type, and sports involvement were collected. **Results:** The majority of patients were male (93.1%), aged 15–30 years (80.2%), and overweight (66.7%). Autografts were the predominant graft choice (96.07%). Associated injuries were present in 79.5% of cases, with medial meniscus ruptures being the most common (37.36%). Sports-related (49.3%) and non-sports-related (50.7%) injuries were nearly equal, with non-contact injuries more prevalent (71.1%). In the sports-related subgroup, associated injuries emerged as a significant risk factor for ACL rupture (*p* = 0.014, OR = 1.596, 95% CI: 1.101–2.314), whereas non-contact mechanisms showed borderline significance (OR = 0.75, *p* = 0.09). Moreover, younger athletes were more susceptible to sports-related injuries (*p* = 0.024), with football being the primary sport involved. **Conclusions:** This study identified a high prevalence of concomitant injuries with ACL injury, which increased the risk of ACL injury, particularly in sports-related cases. Age-related differences in injury patterns highlight the need for age-appropriate preventive measures, especially for younger athletes participating in high-risk sports. This underscores the need for comprehensive injury assessment, targeted prevention strategies, and optimized clinical management approaches tailored to different populations’ specific characteristics and risks.

## 1. Introduction

Anterior cruciate ligament (ACL) injuries are among the most prevalent and debilitating knee injuries [1], with significant adverse effects on a person’s quality of life and long-term joint health [2,3].

The high incidence of ACL injuries, particularly among young athletes and physically active populations, as well as the significant healthcare costs and risk of post-traumatic osteoarthritis associated with these injuries, highlight the importance of comprehensively understanding their epidemiology, risk factors, and management strategies [4,5,6].

Epidemiological research shows that ACL injuries are highly prevalent, with an estimated incidence rate of 68.6 cases per 100,000 person-years in the United States [7,8]. The risk of ACL injury varies according to age, sex, body mass index (BMI), and physical activity level [9,10]. Adolescents, young adults, and females are more likely to suffer from these injuries [5,11]. Moreover, sports involving frequent jumping, pivoting, and cutting maneuvers, such as football and basketball, are associated with an elevated risk of ACL injuries because of the high biomechanical demands placed on the knee joint during these movements [12,13].

ACL injuries are multifactorial and include environmental conditions, competition levels, fatigue, anatomical variations, neuromuscular, and hormonal factors, particularly in females [14,15,16,17].

Surgical reconstruction of the ACL is often recommended for active individuals to return to preinjury activity levels [18]. The choice of graft material (autograft or allograft), presence of associated injuries (e.g., meniscal tears and cartilage lesions), and mechanism of injury can influence surgical decision-making and postoperative outcomes [19,20].

Previous research has highlighted disparities in ACL injury rates across various populations, with distinct patterns observed based on age, sex, body composition, and level of athletic participation [12,21,22]. However, region-specific data are needed to better understand the epidemiology, risk factors, and treatment approaches for ACL injuries across different populations.

This study aimed to comprehensively analyze the epidemiological profile of ACL ruptures in Iranian patients. We focused on demographic factors, injury mechanisms, associated injuries, and surgical management approaches. We hypothesized that specific patient characteristics, such as age, sex, BMI, and sports involvement, would be associated with distinct injury patterns and risk profiles. Furthermore, we aimed to identify potential risk factors for ACL injuries, particularly in the context of sports-related activities. This information can be used to develop targeted prevention strategies and enhance patient care.

## 2. Materials and Methods

### 2.1. Study Design and Setting

This multicenter cross-sectional study retrospectively analyzed the medical records of patients diagnosed with ACL rupture admitted to the Kashani and Al-Zahra Hospitals, the primary trauma referral centers in Isfahan Province, Iran, between October 2016 and December 2020. The Institutional Review Board and Ethics Committee of the Isfahan University of Medical Sciences (IR.MUI.MED.REC.1399.645) approved the study protocol.

### 2.2. Participants

**Figure 1:**
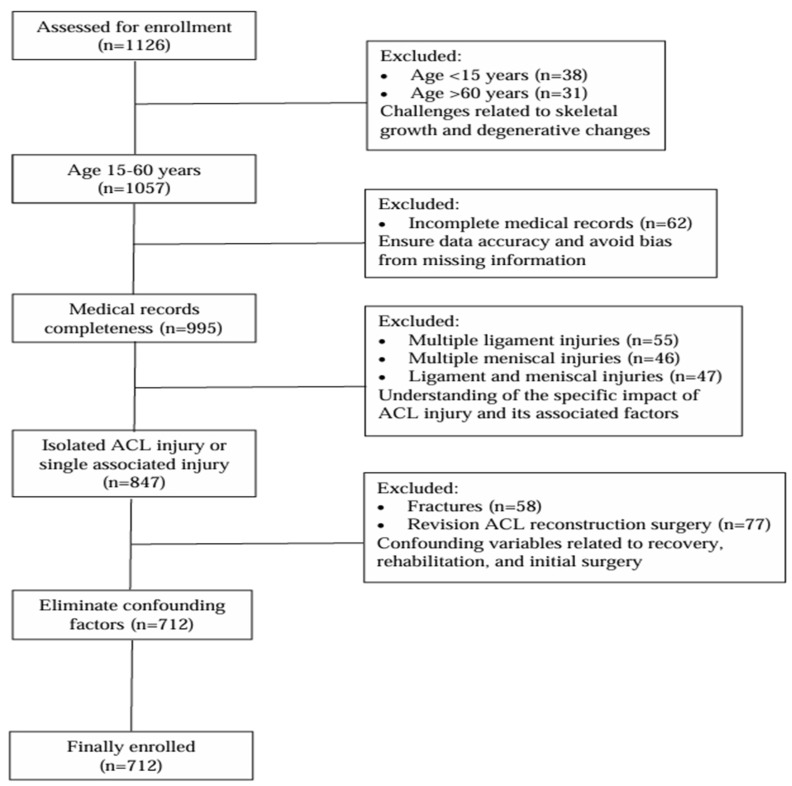
Flow chart outlining the patient selection process and reasons for exclusion.

The study’s participants were carefully selected using census sampling, which thoroughly examined all eligible patient records during the study period. The initial cohort included 1126 patients aged 15 to 60. Exclusions were made for individuals under 15 (*n* = 38) and over 60 (*n* = 31) due to challenges in ACL reconstruction and treatment related to ongoing skeletal development and pre-existing degenerative changes. Additionally, patients with incomplete medical records (*n* = 62) were excluded to ensure data accuracy and avoid bias due to missing information. Multiple ligament injuries (≥two ligaments in addition to ACL) (*n* = 55), multiple meniscal injuries (both medial and lateral menisci) with ACL rupture (*n* = 46), and those with combined meniscal (one or both meniscal) and one or multiple ligament injuries with ACL rupture (*n* = 47) were also excluded. This resulted in a homogenous sample focused solely on isolated ACL injuries or those with only one additional ligament or meniscus injury, providing a clearer understanding of the specific impact of ACL rupture and its associated factors. Patients with fractures (*n* = 58) and revision ACL reconstruction surgery (*n* = 77) were also excluded to eliminate confounding variables related to recovery, rehabilitation, and the initial surgery. The final study sample comprised 712 patients (Figure 1).

### 2.3. Data Collection

The study collected data from patients’ medical records, including demographic details, clinical examination findings, MRI reports, and operative notes. Injuries associated with ACL rupture were identified through MRI reports and intraoperative findings. Based on detailed patient history, injury mechanisms were classified as contact or non-contact. Graft types (autograft or allograft) were obtained from operative notes.

### 2.4. Variables

Age was categorized into three groups: 15–30, 31–45, and 46–60 years, while BMI was classified as underweight <18.5 kg/m^2^, normal 18.5–24.9 kg/m^2^, overweight 25–29.9 kg/m^2^, and obese ≥30 kg/m^2^.

Participants were categorized based on the presence of associated injuries along with the ACL rupture. Those who had injuries to the cartilage, posterior cruciate ligament (PCL), medial collateral ligament (MCL), lateral collateral ligament (LCL), medial meniscus, or lateral meniscus were classified as having associated injuries. Patients who presented only with an isolated ACL rupture, without any concomitant injuries, were categorized as having an isolated ACL injury.

The sports-related injuries occurred during athletic activities, while the non-sports-related injuries occurred during daily activities or non-athletic events. The sports activities leading to ACL injuries included football, running, volleyball, basketball, walking, climbing, wrestling, cycling, and handball.

Injury mechanisms were classified as either non-contact or contact. Non-contact ACL injuries were identified in scenarios without direct external contact or trauma to the knee, typically resulting from the individual’s maneuvers, such as pivoting, landing from a jump, or sudden deceleration, irrespective of the sporting context. Contact ACL injuries occur when an external force is directly exerted on the knee, such as a collision with another participant, equipment, or terrain, or when the knee is subjected to an impact by an object [23,24,25,26]. The mechanism of injury was meticulously determined based on detailed patient history and medical records, enabling accurate classification. The study did not assess potential confounders such as physical activity level and comorbidities due to limitations in the available data.

### 2.5. Statistical Analysis

The study data was analyzed using the SPSS software version 26 (SPSS Inc., Chicago, IL, USA). Descriptive statistics were utilized to summarize demographic characteristics, injury mechanisms, associated injuries, and graft types. Mean ± standard deviation was used for continuous variables, while frequencies and percentages were used for categorical variables. Chi-square tests were used to compare the distribution of categorical variables between groups. A logistic regression analysis was performed to identify potential risk factors for ACL rupture, using odds ratios (ORs) and 95% confidence intervals (CIs) as measures of association. A statistically significant *p*-value of <0.05 was used. Missing data were not imputed, and a complete case analysis was performed.

## 3. Results

### 3.1. Demographic Characteristics

This study included 712 patients with ACL injuries who were referred to our two university referral hospitals. In total, 462 patients were admitted to Kashani Hospital, and 250 were referred to Al-Zahra Hospital.

**Figure 2:**
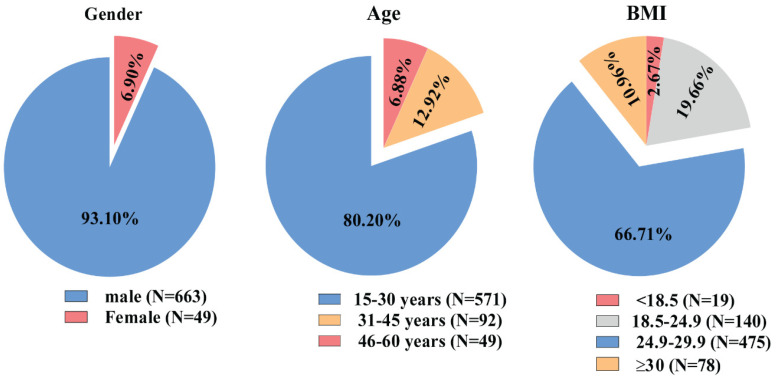
Demographic characteristics of the study participants.

The mean age of the participants was 29.69 ± 7.5 years, within a range of 15 to 60 years. The majority of the patients were male (93.1%), and 80.2% of the participants were in the 15–30 age group. The mean BMI was 26.05 ± 3.2 kg/m^2^, ranging from 16 to 35 kg/m^2^, with 66.7% of the patients classified as overweight (BMI 24.9–29.9 kg/m^2^) (Figure 2).

### 3.2. Injury Characteristics, Surgical Management, and Associated Injuries

**Figure 3:**
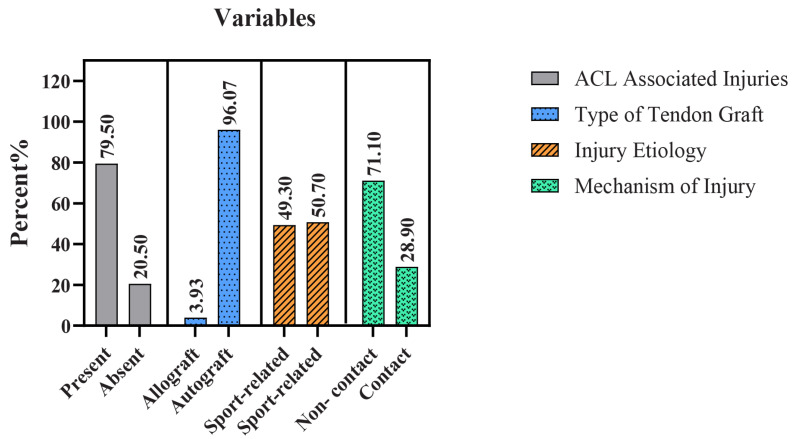
Injury characteristics and surgical management of ACL rupture patients (N = 712).

Figure 3 Summarizes the injury characteristics and aspects of surgical management. Most participants (79.5%, *n* = 566) sustained associated injuries in addition to ACL rupture, whereas 20.5% (*n* = 146) had an isolated ACL injury without the involvement of other knee joint structures. Among the associated injuries, medial meniscus ruptures were the most prevalent, accounting for 37.36%, followed by cartilage injuries and lateral meniscus ruptures, which accounted for 20.51% and 20.08%, respectively. Injuries to the PCL, LCL, and MCL were relatively infrequent, comprising less than 4% combined. Autografts were the predominant choice for ACL reconstruction, utilized in 96.07% (*n* = 684) of cases, whereas allografts were used in only 3.93% (*n* = 28) of patients. Sports-related (49.3%, *n* = 351) and non-sports-related (50.7%, *n* = 361) had nearly equal distribution as etiologies of ACL injury. Noncontact injuries were more prevalent concerning the mechanism of injury (71.1%, *n* = 506) than contact injuries (28.9%, *n* = 206).

### 3.3. Association of Injury Characteristics with Gender

Chi-square analyses were used to investigate the potential associations between injury characteristics and sex (Table 1). No significant differences were observed between males and females regarding the type of tendon graft (*p* = 0.432), mechanism of injury (*p* = 0.19), presence of associated injuries (*p* = 0.58), or sports-related injuries (*p* = 0.30).

### 3.4. Association of Injury Characteristics with Age

The association between injury characteristics and age was examined using the chi-squared test (Table 2). No significant differences were found among the age groups (15–30 years, 31–45 years, and 46–60 years) in the type of tendon graft (*p* = 0.157), mechanism of injury (*p* = 0.66), presence of associated injuries (*p* = 0.137), or sports-related injuries (*p* = 0.90)

### 3.5. Association of Injury Characteristics with BMI

The potential relationship between injury characteristics and BMI was assessed using chi-square analysis (Table 3). No significant differences were observed among the BMI categories (≤18.5, 18.5–24.9, 25–29.9, and ≥30) for the type of tendon graft (*p* = 0.695), mechanism of injury (*p* = 0.122), presence of associated injuries (*p* = 0.084), or sports-related injuries (*p* = 0.791).

### 3.6. Characteristics of Sports-Related ACL Injuries: Analysis by Gender, Age, and BMI

Further analyses were conducted among the subgroup of patients who sustained ACL ruptures during athletic activities (*n* = 351) to gain a deeper understanding of the relationship between demographic factors and the type of sports-related injuries sustained.

Table 4 presents the chi-square analysis of sports-related injuries by sex. No significant differences were found between males and females regarding the type of tendon graft (*p* = 0.269), mechanism of injury (*p* = 0.45), presence of associated injuries (*p* = 0.749), or a specific type of sport leading to the injury (*p* = 0.349).

Table 5 shows the chi-square analysis of sports-related injuries by age group. Significant differences were found among age categories regarding the type of sport (*p* = 0.024), with younger patients (≤30 years) being more likely to sustain ACL injuries during pivoting sports compared to older patients (>30 years). Football was the most common sport associated with ACL injuries. No significant differences were found between the age groups in the type of tendon graft (*p* = 0.529), mechanism of injury (*p* = 0.287), or presence of associated injuries (*p* = 0.115).

Table 6 presents the chi-square analysis of sports-related injuries according to the BMI category. No significant differences were observed among the BMI categories for the type of tendon graft (*p* = 0.735), mechanism of injury (*p* = 0.736), presence of associated injuries (*p* = 0.671), or type of sport leading to the injury (*p* = 0.232).

### 3.7. Risk Factors for ACL Injuries among Athletic Population

Our analysis of the subgroup of patients who sustained ACL ruptures during athletic activities (*n* = 351) revealed risk factors for sports-related ACL injuries (Table 7). The presence of associated injuries emerged as a significant risk factor (*p* = 0.014, OR = 1.596, 95% CI: 1.101–2.314), indicating that patients with a concomitant injury had a higher likelihood of experiencing an ACL rupture during sports compared to those without associated injuries. The mechanism of injury (contact vs. non-contact) showed a borderline significant association (*p* = 0.094, OR = 0.756, 95% CI: 0.545–1.049), suggesting a potential trend towards non-contact injuries being more likely to result in ACL rupture compared to contact injuries. However, other variables such as sex (*p* = 0.192), tendon graft type (*p* = 0.724), BMI (*p* = 0.821), and age (*p* = 0.986) were not found to be significant risk factors for sports-related ACL injuries in this study population.

## 4. Discussion

This comprehensive study provides valuable insights into the epidemiological profile and potential risk factors associated with ACL injuries in a large cohort of Iranian patients. The findings contribute to the growing body of evidence on this crucial topic and underscore the need for targeted preventive strategies and optimized clinical management approaches.

### 4.1. Sex Distribution and Cultural Context

Our study revealed that most ACL injuries occurred in males, accounting for 93.1% of the total cases, with 47.75% being male athletes. Interestingly, this finding contrasts with those of other studies, in which female athletes showed a higher incidence of ACL injuries. In two epidemiological studies, Kaeding et al. [27] reported that female athletes had a relative risk of 1.57 times higher than that of male athletes in comparable sports, such as soccer and basketball. Similarly, Takahashi et al. [28] found that female athletes had a significantly higher incidence of ACL injury than male athletes. In contrast, our study observed a male-to-female athlete ratio of 16.19, which aligns more closely with the Saudi Arabian study, where 98.5% of ACL injuries occurred in males [29]. These findings underscore the importance of considering regional and cultural contexts when analyzing sports-related ACL injuries. They also highlighted the urgent need for interventions tailored to the sex distributions and cultural contexts of the athletes involved. By doing so, we can develop effective injury prevention programs and training practices sensitive to the needs and risks of different groups in different geographic regions, thereby significantly reducing the incidence of ACL injuries.

### 4.2. Graft Selection and Clinical Consideration

Autografts were the preferred choice for ACL reconstruction, accounting for 96.07% of cases, whereas allografts were only utilized in 3.93% of cases. This preference for autografts is frequently observed in clinical practice due to their superior graft incorporation, lower risk of disease transmission, and potential for better clinical outcomes than allografts [30,31]. Nonetheless, the decision regarding the type of graft used may also be influenced by various factors, such as surgeon preference, patient age, and activity level [32,33,34].

### 4.3. Sports-Related vs. Non-Sports-Related ACL Injuries

According to the study, approximately half of all ACL injuries are attributed to sports-related causes (49.3%), whereas the other half are caused by non-sports-related factors (50.7%). This underscores the significance of implementing ACL injury prevention measures in athletic settings, non-sporting activities, and daily life. Previous research has identified several risk factors for non-sports-related ACL injuries, such as occupational activities, inadequate neuromuscular control, and environmental factors [35,36,37,38].

### 4.4. Age-Related Patterns in Sports Injuries

The present study did not find a significant association between age and injury characteristics for the entire cohort, but it provided a more detailed analysis of sports-related injuries, yielding nuanced findings. These findings were significant and highlighted a notable discrepancy between the age groups and type of sport associated with ACL injuries (*p* = 0.024). Football was the most common sport leading to ACL injuries across all age groups, with 234 cases in the 15–30 years group and 35 cases in the 31–45 years group, while the 46–60 age group reported fewer injuries across all sports. This aligns with previous research emphasizing the increased risk of ACL injuries in specific sports and younger age groups [4,8,13].

In addition, our study revealed age-related disparities that could be attributed to various factors. Typically, younger athletes are more prone to participate in high-risk sports such as football, which involves frequent decelerations, changes in direction, and pivoting movements that exert significant stress on the knee joint and ACL [39,40]. Furthermore, neuromuscular immaturity, reduced proprioception, and suboptimal movement patterns, which refer to inefficient or incorrect ways of moving, in younger age groups can contribute to heightened vulnerability to ACL injuries during high-risk activities [15,41].

Our investigation of ACL injuries in athletic populations provided interesting insights. In the sports-related subgroup, we found no significant correlations between age and various injury characteristics, such as tendon graft type, injury mechanism, and associated injuries. This indicates that although age may influence the type of sport resulting in ACL injuries, it may not significantly affect the other characteristics. However, it is crucial to note that the age range of our study participants (80.2% of whom were 15–30 years old) may have limited our ability to identify more subtle age-related disparities. Other factors such as skill level, training routines, and playing environments may also interact with age to alter the risk of ACL injuries in different age groups and sports [42,43,44]. These findings underscore the need to consider age-related patterns and sport-specific demands when developing targeted injury prevention strategies for ACL injury in athletes. Implementing age-appropriate neuromuscular training programs, optimizing movement mechanics, and promoting safe playing environments may be particularly crucial for younger athletes in high-risk sports such as football.

### 4.5. BMI and ACL Injury Risk

The potentially increased risk associated with higher BMI has been attributed to various factors, including increased joint loading, altered biomechanics, and decreased neuromuscular control [45,46]. Individuals with a higher BMI may experience greater ground reaction forces during landing and cutting maneuvers, which could contribute to increased strain on the ACL [47,48]. However, the relationship between BMI and ACL injury risk in athletic populations may also be influenced by other factors such as body composition, muscle strength, neuromuscular control, and biomechanical demands of specific sports [29,30]. While the current study did not find a significant association between BMI and various injury characteristics in the sports-related subgroup, it is essential to note that other studies have reported contrasting findings. For instance, Uhorchak et al. [49] reported that individuals with a BMI greater than 25 kg/m^2^ had a significantly higher risk of ACL injury than those with a lower BMI. Similarly, Labella et al. [45] found that military recruits with body weight or BMI greater than one standard deviation above the mean had 3.2 and 3.5 times higher risks of ACL injury, respectively. The lack of a significant association in our study may be attributed to the BMI distribution in our sample, where the majority (66.71%) were classified as overweight or obese, and the relatively small sample size, which could have limited our ability to detect potential differences across BMI categories, particularly in the higher ranges associated with obesity. Our findings align with some studies that did not observe a significant association between BMI and ACL injury risk [29,50,51]. The relationship between BMI and ACL injury risk remains complex and multifactorial. Further research, including prospective studies with larger sample sizes and comprehensive assessments of body composition and biomechanical factors, is warranted to elucidate the complex interplay among BMI, body composition, biomechanical factors, and ACL injury risk in various athletic populations and sports settings.

### 4.6. Associated Injuries and Clinical Implications

Our research revealed a significant prevalence of associated injuries, with 79.5% of participants having sustained concomitant injuries in addition to ACL rupture, whereas only 20.5% had isolated ACL injuries. Based on our research, medial meniscus rupture was the most common injury observed in ACL tears, accounting for 37.36% of cases. Cartilage injury was the second most prevalent, accounting for 20.51% of cases. These results are consistent with previous studies that have reported a high incidence of meniscal and cartilaginous injuries in association with ACL tears, ranging from 39–65% and 17–43%, respectively [52,53,54,55,56,57].

Bayerl et al. [58] investigated the impact of concomitant medial meniscus injury with ACL injury on individuals. Their findings suggested that patients in such cases experience slightly more discomfort in their everyday lives and face a higher likelihood of developing osteoarthritis ten years post-surgery. The implications of these associated injuries extend beyond the initial trauma and can significantly affect clinical outcomes and the risk of subsequent injuries. Balasingam et al. [59] reported inferior clinical outcomes in patients with concomitant intra-articular injuries compared with those who underwent isolated ACL reconstruction. Moreover, Beynnon et al. [60] found that combined ACL and meniscus injuries were associated with earlier onset and increased rates of post-traumatic osteoarthritis compared with isolated ACL injuries.

### 4.7. Risk Factors for Sports-Related ACL Injuries

Our research found that athletes with additional injuries to knee structures, such as the meniscus, cartilage, collateral ligaments, or PCL, were at a considerably higher risk of experiencing an ACL rupture during sports activities. Individuals who had associated injuries were 1.596 times more susceptible to ACL rupture compared to those who did not have such injuries. Therefore, it is essential to consider the overall health of the knee joint and the cumulative impact of multiple injuries on the risk of ACL rupture among athletes. Our study emphasizes the importance of taking a comprehensive approach to evaluating the knee joint’s health and associated injuries to prevent ACL rupture while participating in sports activities. Paterno et al. [61] discovered that athletes who returned to sports within two years of injury had a greater chance of recurring ACL injury. Shimizu et al. [62] reported that the incidence of reinjury after ACL reconstruction ranged from 3% to 13% in healthy athletic populations. These findings highlight the importance of the potential risk of subsequent injuries and taking appropriate preventive measures. Rehabilitation programs should focus on ACL reconstruction, address concomitant injuries, and optimize overall knee function. Furthermore, educating athletes about the significance of a gradual and safe return to sports following injury is crucial for minimizing reinjury risk.

### 4.8. Mechanism of ACL Injury

Furthermore, the study analyzed the association between the mechanism of injury (contact vs. non-contact) and sports-related ACL injuries and found a borderline significant relationship (*p* = 0.094, OR = 0.756). The results suggest a potential trend toward non-contact injuries being more likely to cause ACL ruptures compared to contact injuries. Non-contact injuries, which usually occur during sudden directional changes, landing, or deceleration maneuvers, put more strain on the ACL and increase the risk of rupture [63,64]. These findings highlight the need for further research to investigate the biomechanical and neuromuscular factors contributing to non-contact ACL injuries in sports.

### 4.9. Limitations and Future Research Directions

Although this study offers valuable insights into the epidemiology and risk factors associated with ACL injuries, it has certain limitations. As this was a retrospective study, it relied on medical records, which may have introduced potential bias or incomplete data. Moreover, the study sample was limited to patients who underwent ACL reconstruction surgery, potentially introducing selection bias and limiting the generalizability of the findings to individuals who received nonsurgical treatment or conservative management. This study did not investigate potential intrinsic risk factors, such as anatomical variations, psychological and neuromuscular deficits, or hormonal factors, which have been implicated in ACL injuries. Including comprehensive assessments of these factors could provide a more thorough understanding of the multifactorial nature of ACL injury. Furthermore, the study did not gather data on return to sports outcomes or distinguish between professional and amateur athletes. Future research should consider prospective study designs with larger and more diverse samples to investigate the long-term outcomes of ACL injuries and to evaluate the effectiveness of targeted prevention programs. Additionally, longitudinal studies examining the effectiveness of targeted prevention programs based on demographic and injury features while exploring the impact of cultural and regional factors on sports participation and injury patterns would provide valuable insights into developing context-specific prevention strategies.

## 5. Conclusions

This comprehensive study provided valuable insights into the epidemiological profile and potential risk factors associated with ACL injuries in a substantial cohort of Iranian patients. These findings highlight the high prevalence of associated injuries, particularly medial meniscus ruptures and cartilage injuries, along with ACL tears. Furthermore, this study identified the presence of associated injuries as a significant risk factor for sports-related ACL injuries, emphasizing the importance of a comprehensive approach to the assessment and management of knee joint health in athletes. Although demographic factors such as age, sex, and BMI did not show a significant association with injury characteristics in the overall cohort, the study found age-related disparities in the type of sports causing ACL injuries, with young male athletes being more susceptible to injuries in high-risk sports involving pivoting and cutting maneuvers, such as football. These findings underscore the need for targeted prevention strategies tailored to specific sports, age groups, and injury patterns, as well as the potential trend toward non-contact injury mechanisms, which suggests the relevance of optimizing neuromuscular training programs and biomechanical assessments in injury prevention programs.

## Figures and Tables

**Table 1:** Association between sex and injury characteristics in ACL rupture patients (N = 712).

Variables	Sub-Categories	Male	Female	*p*-Value
Type of Tendon Grafts	Allograft	25	3	0.432
	Autograft	628	46	
Mechanism of Injury	Non-Contact	467	39	0.19
	Contact	196	10	
ACL Associated Injuries	Yes	525	41	0.58
	No	138	8	
Sport-Leading ACL Injuries	Yes	340	21	0.30
	No	323	28	

**Table 2:** Association between age and injury characteristics in ACL rupture patients (N = 712).

Variables	Sub-Categories	15–30 Years	31–45 Years	46–60 Years	*p*-Value
Type of Tendon Grafts	Allograft	22	6	0	0.15
	Autograft	549	86	49	
Mechanism of Injury	Non-Contact	410	63	33	0.66
	Contact	161	29	16	
ACL Associated Injuries	Yes	447	75	5	0.13
	No	124	17	44	
Sport-Leading ACL Injuries	Yes	288	48	25	0.90
	No	283	44	24	

**Table 3:** Association between BMI and injury characteristics in ACL rupture patients (N = 712).

Variables	Sub-Categories	≤18.5 kg/m^2^	18.5–24.9 kg/m^2^	25–29.9 kg/m^2^	≥30 kg/m^2^	*p*-Value
Type of Tendon Grafts	Allograft	0	7	18	3	0.69
	Autograft	19	133	457	75	
Mechanism of Injury	Non-Contact	10	105	331	60	0.12
	Contact	9	35	144	18	
ACL Associated Injuries	Yes	5	108	374	70	0.08
	No	14	32	101	8	
Sport-Leading ACL Injuries	Yes	9	71	246	38	0.79
	No	11	69	229	40	

**Table 4:** Association between sex and sports-related injury characteristics in ACL rupture patients (N = 351).

Variables	Sub-Categories	Male	Female	*p*-Value
Type of Tendon Grafts	Allograft	13	2	0.269
	Autograft	327	19	
Mechanism of Injury	Non-Contact	252	14	0.45
	Contact	88	7	
ACL Associated Injuries	Yes	283	17	0.749
	No	57	4	
Type of Sport	Basketball	11	0	0.349
	Cycling	3	0	
	Climbing	7	0	
	Football	272	16	
	Handball	3	0	
	Running	15	3	
	Volleyball	15	0	
	Walking	10	1	
	Wrestling	4	1	

**Table 5:** Association between age and sports-related injury characteristics in ACL rupture patients (N = 351).

Variables	Sub-Categories	15–30 Years	31–45 Years	46–60 Years	*p*-Value
Type of Tendon Grafts	Allograft	12	3	0	0.529
	Autograft	276	45	25	
Mechanism of Injury	Non-Contact	215	36	15	0.287
	Contact	73	12	10	
ACL Associated Injuries	Yes	234	42	24	0.115
	No	54	6	1	
Type of Sport	Basketball	8	3	0	0.024 *
	Cycling	1	1	1	
	Climbing	5	1	1	
	Football	234	35	1	
	Handball	1	0	0	
	Running	17	0	1	
	Volleyball	10	4	2	
	Walking	8	3	0	
	Wrestling	4	0	0	

* Significant Variables (*p*-value < 0.05).

**Table 6:** Association between BMI and sports-related injury characteristics in ACL rupture patients (N = 351).

Variables	Sub-Categories	≤18.5 kg/m^2^	18.5–24.9 kg/m^2^	25–29.9 kg/m^2^	≥30 kg/m^2^	*p*-Value
Type of Tendon Grafts	Allograft	0	4	9	2	0.735
	Autograft	8	65	237	36	
Mechanism of Injury	Non- Contact	5	48	184	29	0.736
	Contact	3	21	62	9	
ACL Associated Injuries	Yes	7	58	201	34	0.671
	No	1	11	45	4	
Type of Sport	Basketball	0	4	7	0	0.232
	Cycling	0	1	1	1	
	Climbing	1	1	4	1	
	Football	5	52	201	30	
	Handball	0	1	2	0	
	Running	1	3	13	1	
	Volleyball	0	3	10	2	
	Walking	1	2	7	1	
	Wrestling	0	2	1	2	

**Table 7:** Multivariable logistic regression analysis of risk factors for sports-related ACL injuries (N = 351).

Variables	OR (95% CI)	*p*-Value
Gender	0.674 (0.373–1.219)	0.192
Type of Tendon Grafts	0.871 (0.405–1.873)	0.724
BMI	1.028 (0.811–1.303)	0.821
Age	1.002 (0.775–1.297)	0.986
Type of Injury	0.756 (0.545–1.049)	0.094
ACL Associated Injuries	1.596 (1.101–2.314)	0.014 *

* Significant Variables (*p*-value < 0.05).

## Data Availability

The original data presented in the study are openly available in FigShare at https://doi.org/10.6084/m9.figshare.25706898 (accessed on 26 April 2024).
